# Characteristics of Women of Reproductive Age with Laboratory-Confirmed SARS-CoV-2 Infection by Pregnancy Status — United States, January 22–June 7, 2020

**DOI:** 10.15585/mmwr.mm6925a1

**Published:** 2020-06-26

**Authors:** Sascha Ellington, Penelope Strid, Van T. Tong, Kate Woodworth, Romeo R. Galang, Laura D. Zambrano, John Nahabedian, Kayla Anderson, Suzanne M. Gilboa

**Affiliations:** 1 CDC COVID-19 Emergency Response.

As of June 16, 2020, the coronavirus disease 2019 (COVID-19) pandemic has resulted in 2,104,346 cases and 116,140 deaths in the United States.* During pregnancy, women experience immunologic and physiologic changes that could increase their risk for more severe illness from respiratory infections (*1*,*2*). To date, data to assess the prevalence and severity of COVID-19 among pregnant U.S. women and determine whether signs and symptoms differ among pregnant and nonpregnant women are limited. During January 22–June 7, as part of COVID-19 surveillance, CDC received reports of 326,335 women of reproductive age (15–44 years) who had positive test results for SARS-CoV-2, the virus that causes COVID-19. Data on pregnancy status were available for 91,412 (28.0%) women with laboratory-confirmed infections; among these, 8,207 (9.0%) were pregnant. Symptomatic pregnant and nonpregnant women with COVID-19 reported similar frequencies of cough (>50%) and shortness of breath (30%), but pregnant women less frequently reported headache, muscle aches, fever, chills, and diarrhea. Chronic lung disease, diabetes mellitus, and cardiovascular disease were more commonly reported among pregnant women than among nonpregnant women. Among women with COVID-19, approximately one third (31.5%) of pregnant women were reported to have been hospitalized compared with 5.8% of nonpregnant women. After adjusting for age, presence of underlying medical conditions, and race/ethnicity, pregnant women were significantly more likely to be admitted to the intensive care unit (ICU) (aRR = 1.5, 95% confidence interval [CI] = 1.2–1.8) and receive mechanical ventilation (aRR = 1.7, 95% CI = 1.2–2.4). Sixteen (0.2%) COVID-19–related deaths were reported among pregnant women aged 15–44 years, and 208 (0.2%) such deaths were reported among nonpregnant women (aRR = 0.9, 95% CI = 0.5–1.5). These findings suggest that among women of reproductive age with COVID-19, pregnant women are more likely to be hospitalized and at increased risk for ICU admission and receipt of mechanical ventilation compared with nonpregnant women, but their risk for death is similar. To reduce occurrence of severe illness from COVID-19, pregnant women should be counseled about the potential risk for severe illness from COVID-19, and measures to prevent infection with SARS-CoV-2 should be emphasized for pregnant women and their families.

Data on laboratory-confirmed and probable COVID-19 cases^†^ were electronically reported to CDC using a standardized case report form^§^ or through the National Notifiable Diseases Surveillance System^¶^ as part of COVID-19 surveillance efforts. Data are updated by health departments as additional information becomes available. This analysis includes cases reported during January 22–June 7 with data updated as of June 17, 2020. Included cases were limited to laboratory-confirmed infections with SARS-CoV-2 (confirmed by detection of SARS-CoV-2 RNA in a clinical specimen using a molecular amplification detection test) among women aged 15–44 years from 50 states, the District of Columbia, and New York City. Data collected included information on demographic characteristics, pregnancy status, underlying medical conditions, clinical signs and symptoms, and outcomes (including hospitalization, ICU admission, receipt of mechanical ventilation, and death). Outcomes with missing data were assumed not to have occurred (i.e., if data were missing on hospitalization, women were assumed to not have been hospitalized). Crude and adjusted risk ratios and 95% CIs for outcomes were calculated using modified Poisson regression. Risk ratios were adjusted for age (as a continuous variable), presence of underlying chronic conditions (yes/no), and race/ethnicity. All analyses were performed using SAS (version 9.4; SAS Institute).

During January 22–June 7, among 1,573,211 laboratory-confirmed cases of SARS-CoV-2 infection reported to CDC as part of national COVID-19 surveillance, a total of 326,335 (20.7%) occurred among women aged 15–44 years. Data on pregnancy status were available for 91,412 (28.0%) of these women; 8,207 (9.0%) were pregnant (Table 1). Approximately one quarter of all women aged 15–44 years were aged 15–24 years. A total of 54.4% of pregnant women and 38.2% of nonpregnant women were aged 25–34 years; 22.1% of pregnant women and 38.3% of nonpregnant women were aged 35–44 years. Information on race/ethnicity was available for 80.4% of pregnant women and 70.6% of nonpregnant women. Among pregnant women, 46.2% were Hispanic, 23.0% were non-Hispanic white (white), 22.1% were non-Hispanic black (black), and 3.8% were non-Hispanic Asian compared with 38.1%, 29.4%, 25.4%, and 3.2%, respectively, among nonpregnant women.

**TABLE 1:** Demographic characteristics, symptoms, and underlying medical conditions among women aged 15–44 years with known pregnancy status and laboratory-confirmed SARS-CoV-2 infection (N = 91,412),* by pregnancy status — United States, January 22–June 7, 2020

Characteristic	No. (%)	
	Pregnant women (n = 8,207)	Nonpregnant women (n = 83,205)
**Age group (yrs)**		
15–24	1,921 (23.4)	19,557 (23.5)
25–34	4,469 (54.4)	31,818 (38.2)
35–44	1,817 (22.1)	31,830 (38.3)
**Race/Ethnicity^†^**		
Hispanic or Latino	3,048 (46.2)	22,394 (38.1)
Asian, non-Hispanic	254 (3.8)	1,869 (3.2)
Black, non-Hispanic	1,459 (22.1)	14,922 (25.4)
White, non-Hispanic	1,520 (23.0)	17,297 (29.4)
Multiple or other race, non-Hispanic^§^	321 (4.9)	2,299 (3.9)
**Symptom status^¶^**		
Symptomatic	5,199 (97.1)	72,549 (96.9)
Asymptomatic	156 (2.9)	2,328 (3.1)
Symptom reported**		
Cough	1,799 (51.8)	23,554 (53.7)
Fever^††^	1,190 (34.3)	18,474 (42.1)
Muscle aches	1,323 (38.1)	20,693 (47.2)
Chills	989 (28.5)	15,630 (35.6)
Headache	1,409 (40.6)	22,899 (52.2)
Shortness of breath	1,045 (30.1)	13,292 (30.3)
Sore throat	942 (27.1)	13,681 (31.2)
Diarrhea	497 (14.3)	10,113 (23.1)
Nausea or vomiting	682 (19.6)	6,795 (15.5)
Abdominal pain	350 (10.1)	5,139 (11.7)
Runny nose	326 (9.4)	4,540 (10.4)
New loss of taste or smell^§§^	587 (16.9)	7,262 (16.6)
**Underlying medical condition**		
Known underlying medical condition status^¶¶^	1,878 (22.9)	29,142 (35.0)
Diabetes mellitus	288 (15.3)	1,866 (6.4)
Chronic lung disease	409 (21.8)	3,006 (10.3)
Cardiovascular disease	262 (14.0)	2,082 (7.1)
Chronic renal disease	12 (0.6)	266 (0.9)
Chronic liver disease	8 (0.4)	141 (0.5)
Immunocompromised condition	66 (3.5)	811 (2.8)
Neurologic disorder, neurodevelopmental disorder, or intellectual disability	17 (0.9)	389 (1.3)
Other chronic disease	162 (8.6)	1,586 (5.4)

**Abbreviation:** COVID-19 = coronavirus disease 2019.

* Women with known pregnancy status, representing 28% of 326,335 total cases in women aged 15–44 years.

^†^ Race/ethnicity was missing for 1,605 (20%) pregnant women and 24,424 (29%) nonpregnant women.

^§^ Other race includes American Indian or Alaska Native or Native Hawaiian or Other Pacific Islander.

^¶^ Data on symptom status were missing for 2,852 (35%) pregnant women and 8,328 (10%) nonpregnant women.

** Among symptomatic women (3,474 pregnant; 43,855 nonpregnant) with any of the following symptoms noted as present or absent on the CDC's Human Infection with 2019 Novel Coronavirus Case Report Form: fever (measured >100.4°F [38°C] or subjective), cough, shortness of breath, wheezing, difficulty breathing, chills, rigors, myalgia, rhinorrhea, sore throat, chest pain, nausea or vomiting, abdominal pain, headache, fatigue, diarrhea (three or more loose stools in a 24-hour period), new olfactory or taste disorder, or other symptom not otherwise specified on the form.

^††^ Patients were included if they had information for either measured or subjective fever variables and were considered to have a fever if “yes” was indicated for either variable.

^§§^ New olfactory and taste disorder has only been included on the CDC's Human Infection with 2019 Novel Coronavirus Case Report Form since May 5, 2020. Therefore, data might be underreported for this symptom.

^¶¶^ Status was classified as “known” if any of the following conditions were noted as present or absent on the CDC's Human Infection with 2019 Novel Coronavirus Case Report Form: diabetes mellitus, cardiovascular disease (including hypertension), severe obesity (body mass index ≥40 kg/m^2^), chronic renal disease, chronic liver disease, chronic lung disease, immunosuppressive condition, autoimmune condition, neurologic condition (including neurodevelopmental, intellectual, physical, visual, or hearing impairment), psychological/psychiatric condition, and other underlying medical condition not otherwise specified.

Symptom status was reported for 65.2% of pregnant women and 90.0% of nonpregnant women; among those with symptom status reported, 97.1% of pregnant and 96.9% nonpregnant women reported being symptomatic. Symptomatic pregnant and nonpregnant women also reported similar frequencies of cough (51.8% versus 53.7%) and shortness of breath (30.1% versus 30.3%). Pregnant women less frequently reported headache (40.6% versus 52.2%), muscle aches (38.1% versus 47.2%), fever (34.3% versus 42.1%), chills (28.5% versus 35.6%), and diarrhea (14.3% versus 23.1%) than did nonpregnant women.

Data were available on presence and absence of underlying chronic conditions for 22.9% of pregnant women and 35.0% of nonpregnant women. Chronic lung disease (21.8% pregnant; 10.3% nonpregnant), diabetes mellitus (15.3% pregnant; 6.4% nonpregnant), and cardiovascular disease (14.0% pregnant; 7.1% nonpregnant) were the most commonly reported chronic conditions. Data were not available to distinguish whether chronic conditions were present before or associated with pregnancy (e.g., gestational diabetes or hypertensive disorders of pregnancy).

Hospitalization was reported by a substantially higher percentage of pregnant women (31.5%) than nonpregnant women (5.8%) (Table 2). Data were not available to distinguish hospitalization for COVID-19–related circumstances (e.g., worsening respiratory status) from hospital admission for pregnancy-related treatment or procedures (e.g., delivery). Pregnant women were admitted more frequently to the ICU (1.5%) than were nonpregnant women (0.9%). Similarly, 0.5% of pregnant women required mechanical ventilation compared with 0.3% of nonpregnant women. Sixteen deaths (0.2%) were reported among 8,207 pregnant women, and 208 (0.2%) were reported among 83,205 nonpregnant women. When stratified by age, all outcomes (hospitalization, ICU admission, receipt of mechanical ventilation, and death) were more frequently reported among women aged 35–44 years than among those aged 15–24 years, regardless of pregnancy status. When stratified by race/ethnicity, ICU admission was most frequently reported among pregnant women who were non-Hispanic Asian (3.5%) than among all pregnant women (1.5%) (Table 2).

**TABLE 2:** Hospitalizations, intensive care unit (ICU) admissions, receipt of mechanical ventilation, and deaths among women with known pregnancy status and laboratory-confirmed SARS-CoV-2 infection (N = 91,412), by pregnancy status, age group, and race/ethnicity, and relative risk for these outcomes comparing pregnant women to nonpregnant women aged 15–44 years — United States, January 22–June 7, 2020

Outcome*	No. (%)		Crude risk ratio (95% CI)	Adjusted risk ratio^†^ (95% CI)
	Pregnant women (n = 8,207)	Nonpregnant women (n = 83,205)		
**Hospitalization^§^**			5.4 (5.2–5.7)	5.4 (5.1–5.6)
**All**	2,587 (31.5)	4,840 (5.8)		
**Age group (yrs)**				
15–24	562 (29.3)	639 (3.3)		
25–34	1,398 (31.3)	1,689 (5.3)		
35–44	627 (34.5)	2,512 (7.9)		
**Race/Ethnicity^¶^**				
Hispanic or Latino	968 (31.7)	1,473 (6.5)		
Asian, non-Hispanic	100 (39.4)	136 (7.3)		
Black, non-Hispanic	461 (31.6)	1,199 (8.0)		
White, non-Hispanic	492 (32.4)	803 (4.6)		
Multiple or other race, non-Hispanic**	136 (42.4)	194 (8.4)		
**ICU admission^††^**			1.6 (1.3–1.9)	1.5 (1.2–1.8)
**All**	120 (1.5)	757 (0.9)		
**Age group (yrs)**				
15–24	19 (1.0)	100 (0.5)		
25–34	53 (1.2)	251 (0.8)		
35–44	48 (2.6)	406 (1.3)		
**Race/Ethnicity**				
Hispanic or Latino	49 (1.6)	194 (0.9)		
Asian, non-Hispanic	9 (3.5)	25 (1.3)		
Black, non-Hispanic	28 (1.9)	194 (1.3)		
White, non-Hispanic	12 (0.8)	158 (0.9)		
Multiple or other race, non-Hispanic**	<5 (—^§§^)	40 (1.7)		
Hispanic or Latino	49 (1.6)	194 (0.9)		
**Mechanical ventilation^¶¶^**			1.9 (1.4–2.6)	1.7 (1.2–2.4)
**All**	42 (0.5)	225 (0.3)		
**Age group (yrs)**				
15–24	<5 (—^§§^)	22 (0.1)		
25–34	18 (0.4)	74 (0.2)		
35–44	21 (1.2)	129 (0.4)		
**Race/Ethnicity**				
Hispanic or Latino	13 (0.4)	70 (0.3)		
Asian, non-Hispanic	<5 (—^§§^)	13 (0.7)		
Black, non-Hispanic	9 (0.6)	48 (0.3)		
White, non-Hispanic	<5 (—^§§^)	44 (0.3)		
Multiple or other race, non-Hispanic**	5 (1.6)	16 (0.7)		
Death***			0.8 (0.5–1.3)	0.9 (0.5–1.5)
**All**	16 (0.2)	208 (0.2)		
**Age group (yrs)**				
15–24	<5 (—^§§^)	9 (0.0)		
25–34	7 (0.2)	58 (0.2)		
35–44	8 (0.4)	141 (0.4)		
**Race/Ethnicity**				
Hispanic or Latino	5 (0.2)	47 (0.2)		
Asian, non-Hispanic	<5 (—^§§^)	7 (0.4)		
Black, non-Hispanic	6 (0.4)	74 (0.5)		
White, non-Hispanic	<5 (—^§§^)	37 (0.2)		
Multiple or other race, non-Hispanic**	<5 (—^§§^)	8 (0.4)		

**Abbreviations:** CI = confidence interval; COVID-19 = coronavirus disease 2019.

* Percentages calculated among total in pregnancy status group with known hospitalization status, ICU admission status, mechanical ventilation status, or death.

^†^ Adjusted for age as a continuous variable, dichotomous yes/no variable for presence of underlying conditions, and categorical race/ethnicity variable. Nonpregnant women are the referent group.

^§^ A total of 1,539 (18%) pregnant women and 9,744 (12%) nonpregnant women were missing information on hospitalization status and were assumed to have not been hospitalized.

^¶^ Race/ethnicity was missing for 1,605 (20%) pregnant women and 24,424 (29%) nonpregnant women.

** Other race includes American Indian or Alaska Native or Native Hawaiian or Other Pacific Islander.

^††^ A total of 6,079 (74%) pregnant women and 58,888 (71%) nonpregnant women were missing information for ICU admission and were assumed to have not been admitted to an ICU.

^§§^ Cell counts <5 are suppressed.

^¶¶^ A total of 6,351 (77%) pregnant women and 63,893 (77%) nonpregnant women were missing information for receipt of mechanical ventilation and were assumed to have not received mechanical ventilation.

*** A total of 3,819 (47%) pregnant women and 17,420 (21%) nonpregnant women were missing information on death and were assumed to have survived.

After adjusting for age, presence of underlying conditions, and race/ethnicity, pregnant women were 5.4 times more likely to be hospitalized (95% CI = 5.1–5.6), 1.5 times more likely to be admitted to the ICU ( 95% CI = 1.2–1.8), and 1.7 times more likely to receive mechanical ventilation (95% CI = 1.2–2.4) (Table 2). No difference in the risk for death between pregnant and nonpregnant women was found (aRR = 0.9, 95% CI = 0.5–1.5).

## Discussion

As of June 7, 2020, a total of 8,207 cases of COVID-19 in pregnant women were reported to CDC, representing approximately 9% of cases among women of reproductive age with data available on pregnancy status. This finding is similar to that of a recent analysis of hospitalized COVID-19 patients (*3*); however, given that approximately 5% of women aged 15–44 years are pregnant at a point in time,** this percentage is higher than expected. Although these findings could be related to the increased risk for illness, they also could be related to the high proportion of reproductive-aged women for whom data on pregnancy status was missing, if these women were more likely to not be pregnant. The higher-than-expected percentage of COVID-19 cases among women of reproductive age who were pregnant might also be attributable to increased screening and detection of SARS-CoV-2 infection in pregnant women compared with nonpregnant women or by more frequent health care encounters, which increase opportunities to receive SARS-CoV-2 testing. Several inpatient obstetric health care facilities have implemented universal screening and testing policies for pregnant women upon admission (*4*–*6*). During the study period, among pregnant women with laboratory-confirmed SARS-CoV-2 infection who reported race/ethnicity, 46% were Hispanic, 22% were black, and 23% were white; these proportions differ from those among women with reported race/ethnicity who gave birth in 2019: 24% were Hispanic, 15% were black, and 51% were white.^††^ Although data on race/ethnicity were missing for 20% of pregnant women in this study, these findings suggest that pregnant women who are Hispanic and black might be disproportionately affected by SARS-CoV-2 infection during pregnancy.

Among women with known symptom status, similar percentages of pregnant and nonpregnant women were symptomatic with COVID-19. However, data on symptom status were missing for approximately one third of pregnant women, compared with 10% of nonpregnant women; therefore, if those with missing symptom status are more likely to be asymptomatic, the percentage of pregnant women who are asymptomatic could be higher than the percentage of asymptomatic nonpregnant women. The percentages of pregnant women reporting fever, muscle aches, chills, headache, and diarrhea were lower than those reported among nonpregnant women, suggesting that signs and symptoms of COVID-19 might differ between pregnant and nonpregnant women. Diabetes mellitus, chronic lung disease, and cardiovascular disease were reported more frequently among pregnant women than among nonpregnant women. Additional information is needed to distinguish medical conditions that developed before pregnancy from those that developed during pregnancy and to determine whether this distinction affects clinical outcomes of COVID-19.

Whereas hospitalization occurred in a significantly higher proportion of pregnant women than nonpregnant women, data needed to distinguish hospitalization for COVID-19 from hospital admission for pregnancy-related conditions were not available. Further, differences in hospitalization by pregnancy status might reflect a lower threshold for admitting pregnant patients or for universal screening and testing policies that some hospitals have implemented for women admitted to the labor and delivery unit (*4*–*7*). In contrast, however, ICU admission and receipt of mechanical ventilation are distinct proxies for illness severity (*8*), and after adjusting for age, presence of underlying conditions, and race/ethnicity, the risks for both outcomes were significantly higher among pregnant women than among nonpregnant women. These findings are similar to those from a recent study in Sweden, which found that pregnant women with COVID-19 were five times more likely to be admitted to the ICU and four times more like to receive mechanical ventilation than were nonpregnant women (*9*). The risk for death was the same for pregnant and nonpregnant women. A recent meta-analysis of individual participant data among women of reproductive age found that for influenza, pregnancy was associated with a seven times higher risk for hospitalization, a lower risk for ICU admission, and no increased risk for death (*10*).

The findings in this report are subject to at least four limitations. First, pregnancy status was missing for three quarters of women of reproductive age with SARS-CoV-2 infection. Moreover, among COVID-19 cases in female patients with known pregnancy status, data on race/ethnicity, symptoms, underlying conditions, and outcomes were missing for a large proportion of cases. This circumstance could lead to overestimation or underestimation of some characteristics, if those with missing data were systematically different from those with available data. To avoid overestimating the risk for adverse outcomes, the absence of data on an outcome was assumed to indicate that the outcome did not occur, and those persons with missing information were included in the denominator. Second, additional time might be needed to ascertain and report outcomes such as ICU admission, mechanical ventilation, and death, and this analysis might underestimate the prevalence of these outcomes. Third, information on pregnancy trimester at the time of infection or whether the hospitalization was related to pregnancy conditions rather than for COVID-19 illness was not available and limits the interpretation of hospitalization data. Finally, routine case surveillance does not capture pregnancy or birth outcomes; thus, it remains unclear whether SARS-CoV-2 infection during pregnancy is associated with adverse pregnancy outcomes, such as pregnancy loss or preterm birth.

The findings in this report suggest that among adolescents and women aged 15–44 years with COVID-19, pregnancy is associated with increased risk for ICU admission and receipt of mechanical ventilation, but it is not associated with increased risk for mortality. This report also highlights the need for more complete data to fully understand the risk for severe illness resulting from SARS-CoV-2 infection in pregnant women. Further, collection of longitudinal data for pregnant women with SARS-CoV-2 infection, including information about pregnancy outcomes, is needed to understand the effects of SARS-CoV-2 infection on maternal and neonatal outcomes. To address these data gaps, CDC, in collaboration with health departments, has initiated COVID-19 pregnancy surveillance to report pregnancy-related information and outcomes among pregnant women with laboratory-confirmed SARS-CoV-2 infection. CDC will continue to provide updates on COVID-19 cases in pregnant women. Although additional data are needed to further understand these observed elevated risks, pregnant women should be made aware of their potential risk for severe illness from COVID-19. Pregnant women and their families should take measures to ensure their health and prevent the spread of SARS-CoV-2 infection. Specific actions pregnant women can take include not skipping prenatal care appointments, limiting interactions with other people as much as possible, taking precautions to prevent getting COVID-19 when interacting with others, having at least a 30-day supply of medicines, and talking to their health care provider about how to stay healthy during the COVID-19 pandemic.^§§^ To reduce severe outcomes from COVID-19 among pregnant women, measures to prevent SARS-CoV-2 infection should be emphasized, and potential barriers to the ability to adhere to these measures need to be addressed.

**Summary**

**What is already known about this topic?**

Limited information is available about SARS-CoV-2 infection in U.S. pregnant women.

**What is added by this report?**

Hispanic and non-Hispanic black pregnant women appear to be disproportionately affected by SARS-CoV-2 infection during pregnancy. Among reproductive-age women with SARS-CoV-2 infection, pregnancy was associated with hospitalization and increased risk for intensive care unit admission, and receipt of mechanical ventilation, but not with death.

**What are the implications for public health practice?**

Pregnant women might be at increased risk for severe COVID-19 illness. To reduce severe COVID-19–associated illness, pregnant women should be aware of their potential risk for severe COVID-19 illness. Prevention of COVID-19 should be emphasized for pregnant women and potential barriers to adherence to these measures need to be addressed.

## Footnotes

* https://www.cdc.gov/coronavirus/2019-ncov/cases-updates/cases-in-us.html.

† https://wwwn.cdc.gov/nndss/conditions/coronavirus-disease-2019-covid-19/case-definition/2020/.

§ https://www.cdc.gov/coronavirus/2019-ncov/downloads/pui-form.pdf.

¶ https://wwwn.cdc.gov/nndss/covid-19-response.html.

** https://www.cdc.gov/reproductivehealth/emergency/docs/Geographic-Calculator-for-Pregnant-Women_508.xlsx.

†† https://www.cdc.gov/nchs/data/vsrr/vsrr-8-508.pdf.

§§ https://www.cdc.gov/coronavirus/2019-ncov/need-extra-precautions/pregnancy-breastfeeding.html.
